# A robust detail preserving anisotropic diffusion for speckle reduction in ultrasound images

**DOI:** 10.1186/1471-2164-12-S5-S14

**Published:** 2011-12-23

**Authors:** Xiaoming Liu, Jun Liu, Xin Xu, Lei Chun, Jinshan Tang, Youping Deng

**Affiliations:** 1 College of Computer Science and Technology, Wuhan University of Science and Technology, Wuhan, Hubei, China; 2 Key Laboratory of Molecular Biophysics of the Ministry of Education, College of Life Science and Technology, Huazhong University of Science and Technology, Wuhan, Hubei, China; 3 School of Technology, Michigan Technological University, 1400 Townsend Drive, Houghton, Michigan 49931-1295, USA; 4 Rush University Cancer Center, Rush University Medical Center, Chicago, Illinois 60612, USA

## Abstract

**Background:**

Speckles in ultrasound imaging affect image quality and can make the post-processing difficult. Speckle reduction technologies have been employed for removing speckles for some time. One of the effective speckle reduction technologies is anisotropic diffusion. Anisotropic diffusion technology can remove the speckles effectively while preserving the edges of the image and thus has drawn great attention from image processing scientists. However, the proposed methods in the past have different disadvantages, such as being sensitive to the number of iterations or low capability of preserving the details of the ultrasound images. Thus a detail preserved anisotropic diffusion speckle reduction with less sensitive to the number of iterations is needed. This paper aims to develop this kind of technologies.

**Results:**

In this paper, we propose a robust detail preserving anisotropic diffusion filter (RDPAD) for speckle reduction. In order to get robust diffusion, the proposed method integrates Tukey error norm function into the detail preserving anisotropic diffusion filter (DPAD) developed recently. The proposed method could prohibit over-diffusion and thus is less sensitive to the number of iterations

**Conclusions:**

The proposed anisotropic diffusion can preserve the important structure information of the original image while reducing speckles. It is also less sensitive to the number of iterations. Experimental results on real ultrasound images show the effectiveness of the proposed anisotropic diffusion filter.

## Background

Medical imaging techniques have obtained great development in the past decades and have been found different applications in disease diagnosis. One of these important imaging techniques is ultrasound imaging. ultrasound imaging has many advantages such as noninvasiveness, portability, and low price, which make it attractive to different clinical applications [1]. However, the quality of ultrasound images is greatly affected by speckles, a granular pattern formed due to coherent interferences of backscattered echoes from the scatters [2]. The presence of speckle degrades the quality of ultrasound images, and thus affects diagnosis. Thus, speckle reduction has become an important task in many applications with ultrasound imaging.

Different methods have been investigated for speckle reduction. These methods include early methods such as Lee filter [3], Frost filter [4], Kuan filter [5], and recently developed methods such as adaptive filters [6,7], wavelet transform [8-11], bilateral filters [12], nonlocal-means [13] and anisotropic diffusion filters [14-18], etc. In [6], an adaptive weighted median filter (AWMF) for speckle reduction is proposed. Different from the common median filter, AWMF adjusts weight coefficients and smoothing characteristics based on the local statistics. In [7], an adaptive speckle suppression filter (ASSF) is developed for speckle reduction in B-scan images. The proposed filter used appropriately shaped and sized local filtering kernels and has better adaptation to local variations. In [9], a speckle suppression method is presented for ultrasound images. In the presented method, the original image was first logarithmically transformed, and then 2-D wavelet transform was applied to obtain multiscale decomposition for speckle reduction. Besides the methods described above, anisotropic diffusion filters [14] have been studied deeply in recent years [15-23]. In [15], an anisotropic diffusion method which integrated with the Smallest Univalue Segment Assimilating Nucleus (SUSAN) edge detector was proposed. The proposed method can provide good performance in both speckle reduction and detail preservation. In [16], a nonlinear coherent diffusion (NCD) model for logarithmic compressed B-mode ultrasound images was developed. The proposed method can work in real-time. In [18], Yu *et al. *proposed the speckle reducing anisotropic diffusion (SRAD) method for ultrasonic images. The method integrated spatially adaptive filter into the diffusion technique, and exploited the instantaneous coefficient of variation for edge detection. Compared with previous method, the method has better performance in both edge preservation and speckle reduction. In addition, the SRAD has been further applied to 3D ultrasound images [19,20] and also obtained good performance. Recently, another improvement for anisotropic diffusion filter is the work in [23]. In [23], Tauber *et al. *improved the robustness of the original SRAD by following the analysis of P-M method with respect to the robust estimation of a piecewise smooth image. Inspired by the success of the work [17,23], we will further improve the robustness of the DPAD in this paper.

## Results

In order to test the performance of the proposed method, we have performed several experiments on ultrasound images. The proposed method was compared with the SRAD algorithm [18] developed by Yu and the DPAD algorithm developed by Aja-Fernandez [22].

### Experimental results for speckle reduction

**Figure 1:**
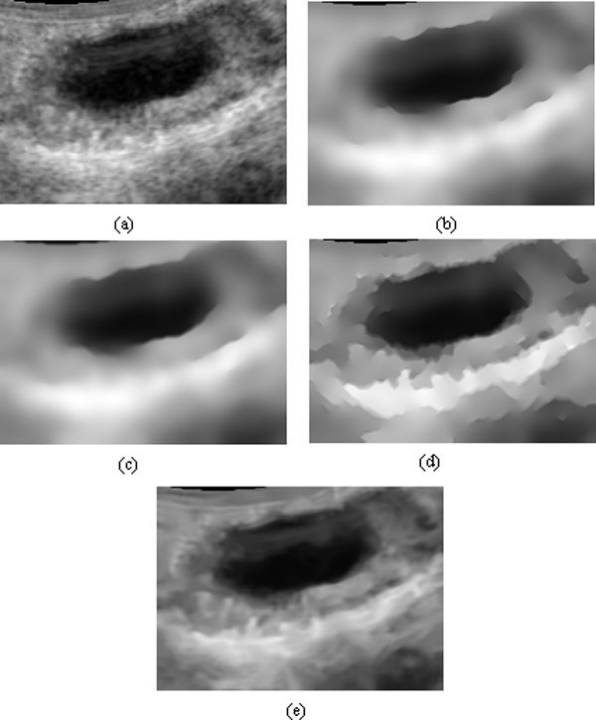
**Experimental results of different methods on a cattle's follicle ultrasound image**. (a) Original image, (b) result with SRAD, (c) result with DPAD, (d) result with RDPAD, (e) result with nonlocal means. The number of iterations is 300 in (b), (c) and (d).

**Figure 2:**
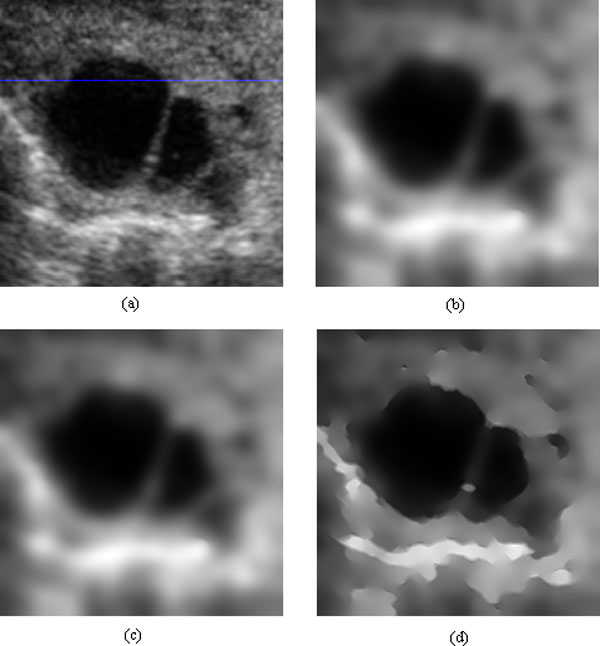
**Experimental results of different methods on another cattle's follicle ultrasound image**. (a) Original image with a line overlapped, (b) result with SRAD, (c) result with DPAD, (d) result with RDPAD. The number of iterations is 300 in (b), (c) and (d).

**Figure 3:**
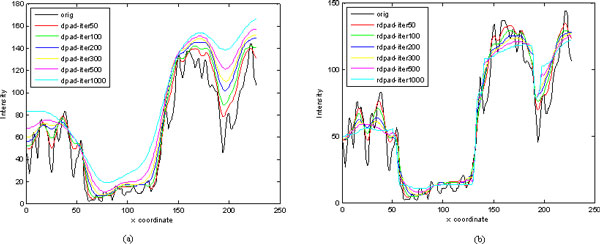
**Experimental results in respect of detail preserving for different methods over a horizontal scan line (row 65) of the ultrasound image in Fig.2 (a)**. (a) Result with DPAD, (b) result with proposed RDPAD.

**Figure 4:**
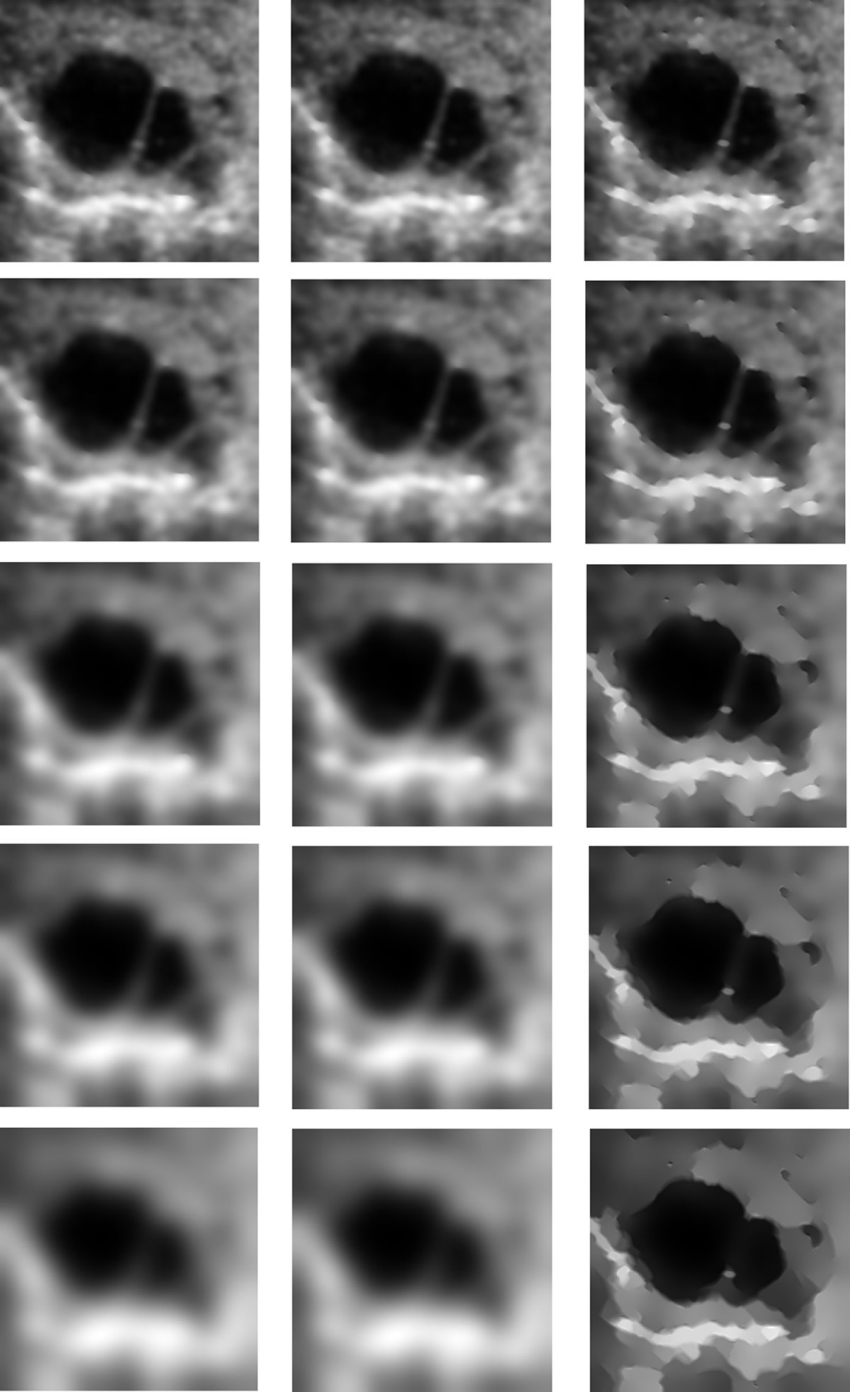
**Results of different methods with respect to the number of iterations on an image shown in Fig.2 (a)**. The first column displays the results obtained by SRAD, the second column displays the results obtained by DPAD, and the third column obtained by for RDPAD. The number of iterations is 50, 100, 300, 500 and 1000 corresponding to rows 1 to 5, respectively.

We performed several experiments to test the performance of the proposed method. In the experiments, the ultrasound images used were from cattle's follicles. Figure 1(a) and Figure 2(a) show two of these original images. Figure 1 and 2's (b), (c) (d) show the experimental results from different methods (SRAD, DPAD, RDPAD). The number of iterations was set to 300. For testing the capability of detail preservation of the proposed method, we compared the pixel values extracted from a blue line as shown in Figure 2(a). Figure 3 shows the intensity values of the blue line after speckle reduction with SRAD and RDPAD when the number of the iteration is 50, 100, 200, 300, 500 and 1000 respectively. Experimental results shown in Figure (3) show that all of these three diffusion methods can reduce the speckles effectively. However, the DPAD doesn't stop diffusion when the number of iterations is increasing. This resulted in smoothed image and many details were lost. The proposed RDAPD method can preserve the details in the diffused image. We also compared our method with the nonlocal-means method. The result obtained by nonlocal-means method for image in Figure 1(a) is shown in Figure 1(e). From the experiments, we also find that nonlocal-means method can also reduce the speckles while preserving some details. However, compared with nonlocal-means method, the proposed method also enhanced the edges. This can also be visually inspected in Figure 4, which shows the diffusion results obtained by different methods with different iteration times. From the experiments, we can find that RDAPD is less sensitive to the number of iterations, which is another advantage of RDAPD over SRAD and DPAD since the number of iterations in diffusion based methods is generally an important parameter.

**Figure 5:**
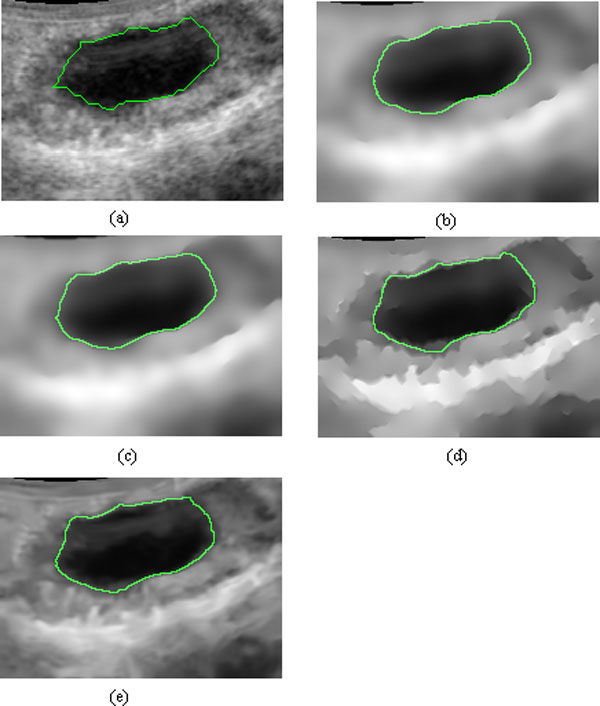
**Segmentation results with different speckle reduction methods**. (a) Original image with manual segmentation, (b) segmentation result with SRAD, (c) segmentation result with DPAD, (d) segmentation result with RDPAD, (e) segmentation result with nonlocal means.

In order to compare the effectiveness of speckle reduction on segmentation, we used active contour without edge (ACWE) developed in [24] to extract the follicle boundaries from ultrasound image. Figure 5 shows the contours of the follicles extracted manually from the original image, and the results extracted by ACWE from the images after speckle reduction with SRAD, DPAD, nonlocal-means, and the proposed method. Figure 5 shows that the final contours obtained from the images pre-processed by SRAD and DPAD are away from the boundary obtained manually while the follicle boundaries obtained from the images pre-processed by nonlocal-means and our RDPAD are closed to the boundary obtained manually. The experimental results show that the proposed method has better performance for speckle reduction.

### Quantitative comparison of speckle reduction methods

For quantitative comparison, we used the measurement developed in [25]. The measurement used in [25] can be used to measure the region contrast of an image. As is known, a better speckle reduction method should preserve edges while reducing speckle. Thus we can use the region contrasts in homogenous regions and edge points before and after speckle reduction to measure the effectiveness of each diffusion method. The region contrast *C_w _*in an image *I *is defined as [25]:

(1) $$C_{w}\left(I\right)=\frac{1}{m}\underset{w}{\sum }c\left(x,y\right)\log\left(1+\left|c\left(x,y\right)\right|\right)$$

where the local contrast at pixel (*x*, *y*), *c*(*x*, *y*) is defined as

**Figure 6:**
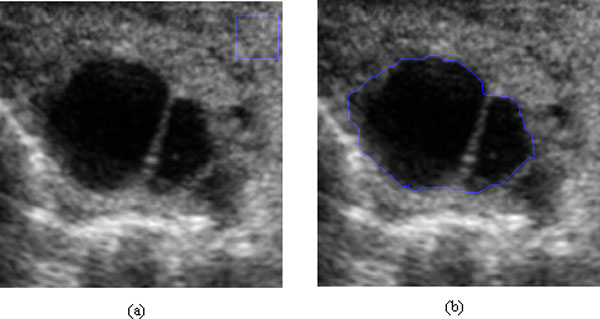
**Homogeneous region and a set of edge points used to calculate RC value**. (a) Homogeneous region, (b) set of edge points.

(2) c(x,y)=4×I(x,y)-{I(x-1,y)+I(x,y-1)+I(x+1,y)+I(x,y+1)}

where *I*(*x*, *y*) is an image pixel intensity value, *w *is a region of image (or a set of points), and *m *is the number of pixels in the region *w *over which the contrast is evaluated. In the experiments, we selected manually a homogeneous region and a set of edge points for measuring the performance of each method, which is shown in Figure 6. Table 1 shows the RC values from the selected homogeneous region and the selected set of edge points. Based on Table 1, SRAD and DPAD can reduce the speckles in the selected homogeneous region effectively, but the CR values of the selected set of edge points are reduced. However, the proposed method can preserve the contrast of the edge points and can remove the speckle in the homogenous region effectively.

**Table 1:** Region contrast (RC) values of different speckle reduction methods

Regions	Original image	SRAD	DPAD	RDPAD
Homogenous region	3.4971	0.0041	0.0041	0.0046
Edge points	2.9330	0.0080	0.0109	2.8597

## Discussion

The proposed speckle reduction can be applied as a preprocessing step for image segmentation [24]. Because ultrasound image segmentation will be affected by speckles, a good speckle reduction method will enhance the performance of image segmentation. Although we have shown some improvement of segmentation after speckle reduction, the number of cases is not big, thus our future work will focus on measuring the performance of speckle reduction on segmentation using large set of ultrasound images.

Another potential application is the extension of the proposed method to 3-D speckle reduction in ultrasound images. As is well known, 3-D ultrasound imaging is a more challenging area than 2-D ultrasound imaging. Based on our current experiments, we predict the proposed method can also get good results for 3-D ultrasound images.

## Conclusion

By integrating the detail preserving anisotropic diffusion developed by Aja-Fernandez and the diffusion coefficient function from [17], we developed a new anisotropic diffusion filter which can have better performance in edge preservation and speckle reduction. Due to the favorable property of "edge-stopping" diffusion, the proposed method is less sensitive to the number of iterations. Experimental results on real ultrasound images indicated that the proposed method can achieve better performance than both SRAD and DPAD. The proposed method provides a preprocessing method for ultrasound image segmentation.

## Methods

### Previous work on anisotropic diffusion for speckle reduction

Anisotropic diffusion was proposed in [14] and has been employed for noise reduction for some time. The basic equation used in anisotropic diffusion is a partial differential equation which can be expressed as [14]:

(3) $$\left\{\begin{array}{c} \frac{\partial I}{\partial t}=div\left[c\left(\left|\nabla I\right|\right)∙\nabla I\right]\\ I\left(t=0\right)=I_{0} \end{array}\right)$$

where ∇ is the gradient operator, *div *is the divergence operator, |•| is the magnitude.

In the study of anisotropic diffusion for speckle reduction, a lot of research focuses on the development of the computation of *c*(*x*). One of the methods is speckle reducing anisotropic diffusion filter developed by Yu and Acton [18]. In [18], they proposed the following equation to compute the diffusion coefficients:

(4) $$c\left(q\right)=\frac{1}{1+\left[q^{2}\left(i,j;t\right)-q_{0}^{2}\left(t\right)\right]/)\left[q_{0}^{2}\left(t\right)\left(1+q_{0}^{2}\left(t\right)\right)\right]}$$

where

(5) $$q{\left(i,j;t\right)}^{2}=\frac{\frac{1}{2}{\left(|\nabla I/)I\right)}^{2}-\frac{1}{16}{\left(\nabla^{2}I/)I\right)}^{2}}{{\left[1+\left(1/)4\right)\left(\nabla^{2}I/)I\right)\right]}^{2}}$$

is called instantaneous coefficient of variation (ICOV).

In fact, SRAD is obtained by combining anisotropic diffusion with Lee filter [22]. Similar to SRAD, Aja-Fernandez et al. developed another anisotropic diffusion filter by combining anisotropic diffusion with Kuan filter. They called their filter as detail preserving anisotropic diffusion (DPAD). DPAD is shown to have similar speckle reduction performance to SRAD but is less sensitive to the diffusion iteration times. DPAD computes the coefficient of variation as follows:

(6) $$q{\left(i,j;t\right)}^{2}=\frac{\frac{1}{\left|\eta_{i,j}^{U}\right|-1}\underset{p\in \eta_{i,j}^{U}}{\sum }{\left(I_{p}-{\bar{I}}_{i,j}\right)}^{2}}{{\bar{I}}_{i,j}^{2}}$$

and the diffusion coefficient function adopted by DPAD is

(7) $$c\left(q\right)=\frac{1+\frac{1}{q{\left(i,j;t\right)}^{2}}}{1+\frac{1}{q_{0}{\left(t\right)}^{2}}}$$

Besides Aja-Fernandez's work, Tauber et al. [23] modified the diffusion in SRAD and used:

(8) $$c\left(q\right)=\left\{\begin{array}{cc} \frac{1}{2}{\left[1-\frac{q{\left(i,j;t\right)}^{2}-q_{0}{\left(t\right)}^{2}}{q_{0}{\left(t\right)}^{2}\left(1+q_{0}{\left(t\right)}^{2}\right)}\right]}^{2} & \text{if }\frac{q{\left(i,j;t\right)}^{2}-q_{0}{\left(t\right)}^{2}}{q_{0}{\left(t\right)}^{2}\left(1+q_{0}{\left(t\right)}^{2}\right)}\leq 1\\ 0 & \text{otherwise} \end{array}\right)$$

as the diffusion coefficient function. He used the same way as SRAD to compute the coefficient of variation but different diffusion coefficient function. The diffusion coefficient function in (8) is from [17]:

(9) $$c\left(x,\sigma_{e}\right)=\left\{\begin{array}{cc} \frac{1}{2}{\left[1-{\left(x∕\sigma_{e}\right)}^{2}\right]}^{2} & \left|x\right|\leq \sigma_{e}\\ 0 & \text{otherwise} \end{array}\right)$$

The diffusion coefficient function in (9) allows the neighbours with larger gradient magnitude than *σ_e _*has no influence on the current pixel. The method can preserve sharper edges than previous formulations.

Inspired by their success [17,22,23], in this paper, we aim to improve the robustness of DPAD algorithm and develop a modified algorithm with both advantages from DPAD and Tauber' algorithm [23]. The modified algorithm will preserve sharper edges and be less sensitive to the iteration times.

### The proposed robust detail preserving anisotropic diffusion

In this section, we will develop a new scheme to compute the instantaneous coefficient of variation, and then we introduce the new technique which combines the DPAD algorithm and the diffusion coefficient function in equation (9) from [17]. The proposed method will have the advantages of being robust to outliers (the edges of the image) and less sensitive to the number of diffusion iterations.

#### Computation of instantaneous coefficient of variation with a new scheme

**Figure 7:**
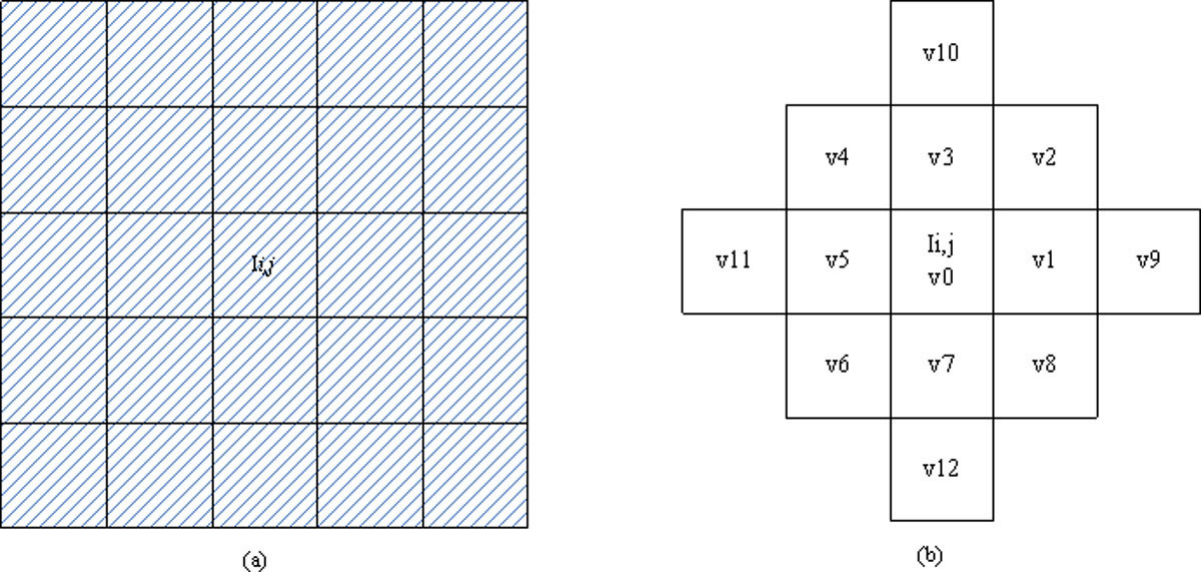
**Estimation windows**. (a) 5 × 5 square window used by Aja-Fernandez et al. (b) Modified 5 × 5 window used by the proposed RDPAD.

In SRAD and DPAD, coefficient of variation is adopted to distinguish homogeneous regions from edges. However, the computation of coefficient of variation from 3 × 3 neighbour is not robust [21], and thus DPAD adopted 5 × 5 neighbour (as shown in Figure 7(a)) to compute *q*(*i*, *j*;*t*). However, the computation using 5 by 5 neighbours is a little costive. In order to make the diffusion robust and less costive, we propose a new scheme to compute *q*(*i*, *j*;*t*). The new scheme is shown in Figure 7(b). Let the pixels be ***v***0,.. ***v***_12 _as shown in the Figure 7, (8) can be reformulated as:

(10) $$q{\left(i,j;t\right)}^{2}=\frac{\frac{1}{12}\overset{11}{\underset{m=0}{\sum }}\overset{12}{\underset{n=m+1}{\sum }}{\left(v_{n}-v_{m}\right)}^{2}}{{\left(\overset{12}{\underset{m=0}{\sum }}v_{m}\right)}^{2}}$$

#### Robust DPAD diffusion function (RDPAD)

Now let's introduce robust DPAD (RDPAD). Starting from equation (9), we have:

(11) $$\begin{array}{lll} c\left(q\right) & =\frac{1+\frac{1}{q{\left(i,j;t\right)}^{2}}}{1+\frac{1}{q_{0}{\left(t\right)}^{2}}}=\frac{q_{0}{\left(t\right)}^{2}\left[1+q{\left(i,j;t\right)}^{2}\right]}{q{\left(i,j;t\right)}^{2}\left[1+q_{0}{\left(t\right)}^{2}\right]}\; & \text{(1)}\\ & =\frac{1}{\frac{q{\left(i,j;t\right)}^{2}+q{\left(i,j;t\right)}^{2}q_{0}{\left(t\right)}^{2}}{q_{0}{\left(t\right)}^{2}+q_{0}{\left(t\right)}^{2}q{\left(i,j;t\right)}^{2}}}=\frac{1}{1+\frac{q{\left(i,j;t\right)}^{2}-q_{0}{\left(t\right)}^{2}}{q_{0}{\left(t\right)}^{2}\left[1+q{\left(i,j;t\right)}^{2}\right]}}\; & \text{(2)}\\ & \; & \text{(3)} \end{array}$$

Let

(12) $$R\left(i,j;t\right)=\frac{q{\left(i,j;t\right)}^{2}-q_{0}{\left(t\right)}^{2}}{q_{0}{\left(t\right)}^{2}\left[1+q{\left(i,j;t\right)}^{2}\right]}$$

Using equation (9) and equation (12), we can obtain a new computation of c(q), which can be expressed as follows:

(13) $$c\left(q\right)=\left\{\begin{array}{cc} \frac{1}{2}{\left[1-\frac{q{\left(i,j;t\right)}^{2}-q_{0}{\left(t\right)}^{2}}{q_{0}{\left(t\right)}^{2}\left[1+q{\left(i,j;t\right)}^{2}\right]}\right]}^{2} & \text{if }\frac{q{\left(i,j;t\right)}^{2}-q_{0}{\left(t\right)}^{2}}{q_{0}{\left(t\right)}^{2}\left[1+q{\left(i,j;t\right)}^{2}\right]}\leq 1\\ 0 & \text{otherwise} \end{array}\right)$$

The above equation can be rewritten as

(14) $$c\left(q\right)=\left\{\begin{array}{cc} \frac{1}{2}{\left[1-\frac{q{\left(i,j;t\right)}^{2}-q_{0}{\left(t\right)}^{2}}{q_{0}{\left(t\right)}^{2}\left[1+q{\left(i,j;t\right)}^{2}\right]}\right]}^{2} & \text{if }q{\left(i,j,t\right)}^{2}\leq \frac{2q_{0}{\left(t\right)}^{2}}{\left|1-q_{0}{\left(t\right)}^{2}\right|}\\ 0 & \text{otherwise} \end{array}\right)$$

In equation (14), we assigns zero weights to the outliers (edges can be seen as outliers in an image) when the instantaneous coefficients of variation is larger than$\frac{2q_{0}{\left(t\right)}^{2}}{\left|1-q_{0}{\left(t\right)}^{2}\right|}$. However, a decreasing small positive weight is assigned to outliers in Aja-Fernandez's algorithm. Therefore, although both of the proposed method and Aja-Fernandez's method perform diffusion similarly when *q *is small. The behaviour of the two methods will be different when q is large. In the case of large q, the proposed method will stop diffusion while Aja-Fernandez will still perform diffusion. Thus the proposed method can result in sharper edges than Aja-Fernandez's method and the proposed method is also robust to the diffusion iterations.

The proposed anisotropic diffusion can be implemented numerically using the similar way to SRAD, the only difference lies in that the computation of c(q) is different.

## Competing interests

The authors declare that they have no competing interests.

## Authors' contributions

XL, JL, LC, XX and JT were involved in the methods design. XL, JL, LC were involved with methods development, coordination and data collection. XL, XX, LC and YD were involved with data analysis. XL, JL, LC, XX are responsible for the writing of manuscript and JT revised some parts of the paper based on the original paper.
